# Alzheimer's Disease Trajectory and the In Vivo Anatomy of Tau Progression

**DOI:** 10.1002/alz70856_099494

**Published:** 2025-12-24

**Authors:** Keith A. Johnson

**Affiliations:** ^1^ Massachusetts General Hospital, Harvard Medical School, Department of Neurology, Boston, MA, USA

## Abstract

**Background:**

Tau PET enables longitudinal tracking of the trajectory of AD tauopathy with respect both to signal intensity and anatomic location. Typically beginning in a specific, small area of the medial temporal lobe (MTL), the rhinal cortex, deposition of tau fibrils increases with increasing amyloid‐β (Aβ) burden at first slowly then catastrophically to neocortex in association with cognitive decline. However, both the anatomy and PET signal intensity are heterogeneous across individuals, with some showing, e.g., a more rapid rise in tau deposition at lower levels of amyloidosis.

**Method:**

We have been able to characterize such heterogeneity in two longitudinal studies of pre‐clinical AD, the Harvard Aging Brain Study (HABS; mean(sd) follow‐up duration 3.3(2.3)yr) and the A4 study (3.3(1.8)y).

**Result:**

Among those with Aβ >24CL (*N* = 229), those who also had tau > 1.2 SUVr (*N* = 112, 49%) had increasing tau; below this tau threshold, spread of tau PET signal to neocortical regions was not detected during the follow‐up interval (slope ∼0).

**Conclusion:**

These analyses may be useful for clinical trial enrollment targeting specific phases of the AD tau progression trajectory.

